# The intricate interplay between dietary habits and cognitive function: insights from the gut-brain axis

**DOI:** 10.3389/fnut.2025.1539355

**Published:** 2025-01-29

**Authors:** Ruyi Zhang, Meiya Zhang, Pengyu Wang

**Affiliations:** ^1^School of Pharmacy, Hubei University of Chinese Medicine, Wuhan, China; ^2^Basic Medical School, Xianning Medical College, Hubei University of Science and Technology, Xianning, China; ^3^School of Pharmacy, Xianning Medical College, Hubei University of Science and Technology, Xianning, China

**Keywords:** cognitive function, gut-brain axis, Mediterranean diet, plant-based diet, low-carbohydrate diet, neuroprotective nutrients

## Abstract

The intricate relationship between dietary habits and cognitive function is gaining increasing attention, with a focus on the gut-brain axis as a modifiable target for intervention. This review synthesizes evidence on the impact of dietary patterns, particularly the Mediterranean diet, plant-based diets, and low-carbohydrate diets, on cognitive health. These diets, rich in antioxidants, anti-inflammatory compounds, and neuroprotective nutrients, are suggested to slow cognitive decline and reduce the risk of neurodegenerative disorders through mechanisms such as reduced inflammation and oxidative stress, and enhanced neurogenesis. The Mediterranean diet has been associated with improved cognitive performance and a delay in cognitive decline in elderly populations. However, challenges in dietary intervention implementation, including adherence and individual variability, remain. Future research must adopt a multidisciplinary approach, incorporating long-term, large-scale, multicenter randomized controlled trials to assess the enduring impacts of various dietary patterns on cognitive function, considering socioeconomic and cultural factors. This review underscores the potential of dietary interventions to prevent and mitigate cognitive impairment, ultimately aiming to improve quality of life.

## Graphical abstract

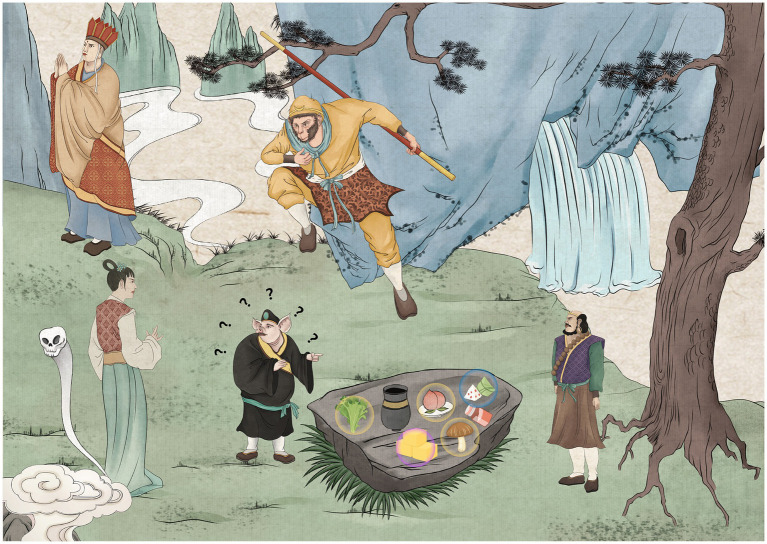

## 1 Introduction

Cognitive health serves as a fundamental component of overall well-being and is increasingly recognized as a critical factor influencing quality of life, particularly among aging populations (1). The World Health Organization (WHO) projects that by 2050, the global demographic of individuals aged 60 and above will reach 2.2 billion, constituting 22% of the world’s population (2). As the demographic shifts toward older age groups, we are witnessing a rise in cognitive decline and the incidence of dementia, which present significant challenges for healthcare systems and society at large on a global scale. Notably, Alzheimer’s disease (AD) and various forms of dementia have emerged as pressing public health concerns worldwide. The 2019 Global Dementia Report by Alzheimer’s Disease International indicates that the number of individuals living with dementia is anticipated to escalate from 50 million in 2019 to 152 million by 2050 (3, 4). This surge not only profoundly affects patients and their families but also exerts substantial strain on healthcare resources and the economy. Consequently, the identification of modifiable risk factors and the development of effective interventions to enhance cognitive health have become paramount public health objectives.

The gut-brain axis, a bidirectional communication system linking the central nervous system with the enteric nervous system, it can use the microorganisms in the intestine to influence the signal transmission of the enteric nervous system, thereby directing brain activity and behavior, and play a pivotal role in cognitive function (5). An imbalance in the gut microbiota, a key component of this axis, has been associated with an increased risk of cognitive impairment. Studies have shown that intestinal microorganisms can affect the regulation of the gut-brain axis through the nervous system, endocrine system, immune system, and metabolic pathways (6). The nervous system transmits information to the brain, the endocrine system is responsible for growth and metabolism, the immune system protects the body from harm, and glands secrete hormones into the blood for communication. The gut microbiota influences cognitive processes through the production of neurotransmitters, modulation of inflammation, and regulation of the blood–brain barrier (7–9). Therefore, understanding the interplay between the gut-brain axis and cognitive health is essential for developing novel therapeutic strategies.

Dietary patterns have long been hypothesized to influence cognitive function, with a growing body of evidence supporting this relationship. The Mediterranean diet (MeDi), plant-based diets, and low-carbohydrate diets (LCDs) have garnered attention for their potential neuroprotective effects (10–12). MD was initially proposed by Keys et al. with the goal of promoting healthy aging and reducing the risk of disease through dietary modifications (13). Today, it is widely recognized as one of the healthiest dietary patterns globally. Plant-based diets generally refer to dietary patterns centered around plant-derived foods while including minimal or no animal-based products. However, different researchers hold varying definitions of plant-based diets. Notably, Satija et al. introduced the plant-based diet index (PDI), which has become a widely accepted criterion for evaluating adherence to plant-based diets (14). LCDs focus on significantly reducing carbohydrate intake while increasing the proportion of protein and fat. The LCD scoring system is currently regarded as the standard metric for assessing adherence to such diets (15). These dietary patterns are characterized by high consumption of fruits, vegetables, whole grains, nuts, and healthy fats, which are rich in bioactive compounds with antioxidant and anti-inflammatory properties. Conversely, excessive intake of saturated fats, sugars, and processed foods has been associated with an increased risk of cognitive impairment. The purpose of this review is to synthesize the latest evidence on the impact of dietary interventions on cognitive health, with a focus on the Mediterranean diet, plant-based diets, and low-carbohydrate diets. We aim to critically evaluate the mechanisms by which these dietary patterns may confer cognitive benefits, including their effects on inflammation, oxidative stress, and neurogenesis.

Furthermore, we will examine the role of specific nutrients, such as polyunsaturated fatty acids, B vitamins, polyphenols, and vitamin D, in cognitive protection (16, 17). The aim of this review is to offer an exhaustive overview of the existing research, which is targeted at guiding clinical practice and public health strategies for preventing cognitive decline and improving the quality of life of people prone to cognitive impairment. Moreover, it intends to further explore the potential role of dietary interventions along the gut-brain axis in promoting cognitive function and alleviating symptoms of neurodegenerative diseases. It presents a comprehensive management strategy for cognitive health and aging.

## 2 Analysis of the correlation between diet and cognitive dysfunction

### 2.1 Mediterranean diet

The “Mediterranean Diet” has its roots in the “Seven Countries Study” initiated by Ancel Keys in the early 1960s, and it was formally recognized at the International Mediterranean Diet Conference in 1993 (18). The MeDi is characterized by a plant-based dietary pattern with a significant emphasis on extra virgin (cold-pressed) olive oil, a variety of vegetables—especially leafy greens—fruits, whole grains, nuts, and legumes. This dietary framework also includes a moderate intake of fish, other meats, dairy products, and red wine, while limiting the consumption of eggs and sugars. Studies had shown that the MeDi could reduce the *Firmicutes*-to-*Bacteroidetes* ratio and increase the levels of SCFAs in feces (19).

It is widely recognized that the MeDi constitutes a beneficial dietary framework. An epidemiological investigation revealed that a diet incorporating a variety of nutrients is more efficacious in mitigating the risk of Alzheimer’s disease (AD) and enhancing cognitive function compared to an emphasis on singular nutrients (20). The study indicates that the health advantages associated with the MeDi may arise from elevated consumption of monounsaturated fats and polyphenols found in olive oil, polyunsaturated fats sourced from fish, as well as the antioxidant characteristics present in vegetables, fruits, and wine (21). In comparison to alternative dietary frameworks, the MeDi demonstrates superior scalability. A study conducted by GUADALUPE revealed that interventions based on the MeDi were linked to a decrease in age-related cognitive decline in Chile, a nation characterized by a climate akin to that of the Mediterranean region (22). A longitudinal investigation conducted by CINTA found that among local elderly cohorts in the U.S., France, Spain, and Greece, adherence to the Mediterranean diet, particularly when enhanced with olive oil or nuts, was associated with improved cognitive performance (23).

Research has established a positive correlation between the MeDi and cognitive performance in the elderly. The diet can delay the progression of cognitive decline associated with AD and vascular dementia, providing significant preventative measures even before clinical onset (24). A fundamental aspect of the MeDi is the endorsement of extra virgin olive oil (EVOO) as the principal source of fat. The nutritional properties of EVOO are vital in mitigating age-associated cognitive deterioration and cognitive deficits. A study conducted by OLIVERAS et al. revealed that individuals participating in the MeDi intervention who incorporated high-polyphenol EVOO into their daily regimen exhibited enhanced antioxidant levels, which contribute to the prevention of additional cognitive decline in patients with Alzheimer’s disease (25). Comparable findings were also corroborated by a prospective cohort study carried out by Tsolaki et al. (26). Moreover, moderate wine intake is a significant characteristic of the MeDi. A randomized controlled trial conducted by RESTANI revealed that the wine suggested in MeDi frequently outperforms other alcoholic drinks in improving cognitive function (27). Furthermore, a double-blind controlled study conducted by Lee et al. revealed that moderate wine intake, when incorporated into a standard diet, provides protective benefits against recognized pathological metabolic deterioration in the early stages of Alzheimer’s disease (28). Subsequently, dairy products represent another key component of the MeDi pattern. The beneficial outcomes associated with dairy interventions may stem from alterations in the ratio of omega-3 to omega-6 fatty acids, which can diminish the synthesis of inflammatory mediators, consequently affecting age-related cognitive decline and the risk of AD. A study conducted by Talaei et al. indicates that dairy consumption during middle adulthood may confer protective benefits against cognitive deterioration (29). Vegetables and fruits are fundamental elements of the MeDi and are vital for maintaining cognitive health. The compounds that enhance cognitive function in these foods are probably not isolated entities; instead, they stem from the synergistic effects of a diverse array of antioxidant nutrients and non-nutritive bioactive compounds found within them.

A cohort study conducted by Wu demonstrated that greater adherence to the MeDi correlates with a more significant delay in cognitive decline and a reduced risk of AD when compared to lower adherence levels (30). Mediterranean Diet interventions play a crucial role in enhancing cognitive function in AD and in delaying its onset. These interventions offer numerous benefits, such as cost-effectiveness, ease of implementation, and minimal observable side effects and contraindications. Consequently, the Mediterranean Diet should be regarded as a fundamental approach in the prevention and management of metabolic syndrome (MetS).

### 2.2 Plant-based diets

In recent years, motivated by the imperative of animal welfare, ecological sustainability, and the enhancement of human health, the academic community has advocated for a decrease in the intake of animal-derived foods within human diets to facilitate a shift toward more healthful dietary practices (31). Plant-based foods, including vegetables, fruits, whole grains, legumes, nuts, and seeds, are essential elements of nutrition, with plant-based diets that minimize or restrict animal-derived products such as meat, eggs, and dairy gaining traction as a prominent dietary trend in affluent nations (32, 33). Plant-based dietary approaches encompass pescatarian, lacto-ovo-vegetarian, vegan, and various other plant-centric eating patterns, which are recognized as primary strategies for preventing or possibly postponing cognitive decline (34, 35). Emerging research indicates that diets rich in plant-based foods contribute positively to neurological well-being (36, 37). Wu found that a plant-based diet during middle age is associated with a reduced risk of cognitive impairment in later years (38). A substantial cohort study conducted in Taiwan, involving 12,062 participants, revealed that vegetarians exhibited a 38% reduced risk of developing dementia in comparison to their non-vegetarian counterparts. Conversely, a cohort study comprising 13,588 healthy adults in the United States found no significant correlation between high meat consumption (OR = 1.06, [95% CI: 0.92, 1.22], *p =* 0.88) or elevated fruit and vegetable intake (OR = 0.99, [95% CI: 0.88, 1.12], *p =* 0.34) and the incidence of dementia after a 20-year follow-up period, which may be closely associated with the duration of the follow-up (39, 40). Furthermore, studies suggest that plant-based diets can influence depressive symptoms among the elderly population. A higher intake of nutritious plant-based foods is associated with a decreased risk of depression, whereas an increased consumption of less healthy plant-based elements correlates with an elevated risk (41). Subsequent research indicates that suboptimal plant-based dietary patterns exert a more pronounced effect on depressive symptoms among elderly individuals exhibiting central obesity, in contrast to their counterparts without such conditions (42). Consequently, dietary patterns centered around plant-based foods may have a beneficial impact on cognitive function.

The pathways through which plant-based dietary patterns influence cognitive health encompass the following: (1) Mitigating the risk of cardiometabolic disorders: Following a nutritious diet and maintaining optimal weight management can substantially lower the risk of cardiometabolic conditions, which serve as precursors to cognitive decline (43, 44). Extensive research has demonstrated that vegetables, fruits, and plant-based oils are abundant in essential nutrients, including polyphenols, unsaturated fatty acids (such as Omega-3 and Omega-6), and dietary fiber. These nutrients have been shown to mitigate inflammation and oxidative stress, thereby impacting the development of neurodegenerative disorders (45–50). Flavonoid modulation of inflammation and oxidative stress: Dietary flavonoids have the capacity to modulate systemic inflammation and oxidative stress, thereby affecting metabolites associated with the gut-microbiota-brain axis. Most of the research indicates a consistent inverse correlation between flavonoid consumption and cognitive performance. Furthermore, unsaturated fatty acids play a crucial role in the regulation of metabolism, immune responses, and inflammatory mechanisms within the central nervous system (51, 52). (2) Supporting the gut microbiota: Plant-based diets may benefit the gut microbiota, directly affecting neurotransmitters and acting as part of the gut-brain axis. Consuming tryptophan-rich foods can elevate 5-HT (serotonin) levels in the body, contributing to improved mood regulation. Additionally, the probiotic strain *Bifidobacterium longum infantis 35,624* has been shown to modulate the 5-HT pathway, thereby reducing depressive-like behaviors (53, 54). The gut microbiota plays a significant role in enhancing the breakdown of fiber and polyphenols while suppressing the breakdown of bile acids, choline, L-carnitine, and amino acids. These mechanisms may have implications for the central nervous system (55–57).

Recent findings indicate that plant-based diets offer protective benefits for cognitive health and related risk factors. It is crucial to select nutritious plant-based foods to maximize these advantages. Thus, dietary recommendations should contemplate integrating wholesome plant-based dietary patterns as a viable approach to address and prevent cognitive health issues.

### 2.3 Low-carbohydrate diets

Low-carbohydrate diets are characterized by limiting carbohydrates to less than 20% of total caloric intake (58). This involves reducing high-carbohydrate foods like sugars, bread, and pasta, while increasing the intake of fats and proteins such as meat, poultry, fish, eggs, cheese, nuts, and seeds (59).

LCDs are a dietary strategy aimed at reducing food consumption without causing malnutrition (60). Research in rodent models has demonstrated that cutting caloric intake by 20–40% can extend lifespan and improve cognitive function, making LCDs one of the most effective nutritional approaches for enhancing cognition in rodents (61). Studies dating back to the mid-1930s have consistently shown the cognitive benefits of LCDs in mouse models, with further research in humans indicating that promoting calorie restriction can help combat age-related cognitive decline and cognitive impairments associated with obesity and type 2 diabetes (62–69).

The mechanisms by which LCDs benefits cognitive health include the following: (1) Reduction of Oxidative Stress: Research indicates that oxidative stress contributes to the high metabolic activity of the brain, particularly affecting regions involved in cognitive functions such as the frontal cortex, amygdala, and hippocampus. LCD’s antioxidant properties play a critical role in protecting neurons by regulating ROS production and maintaining internal balance (70–73). (2) Promotion of Anti-inflammatory Responses: LCDs is linked to anti-inflammatory effects that are crucial for cognitive health. Inflammation, associated with cellular aging, can impact neuron survival and connectivity. LCDs has been shown to reduce inflammation by suppressing the activation of astrocytes and enhancing neuroprotective factors (73–75). (3) Enhancement of Neurogenesis and Synaptic Plasticity: Neurogenesis, the generation of new cells from neural progenitor cells, is essential for learning, memory consolidation, and tissue repair. BDNF, a key neurotrophic factor, influences neurogenesis and synaptic plasticity, thereby affecting cognitive performance. LCDs has the potential to increase BDNF levels in the hippocampus and prefrontal cortex, enhancing spatial and working memory (76–80).

In conclusion, LCDs can positively influence cognition through various pathways. Further research is needed to understand the intricate interactions of these mechanisms within the complex structure and functions of the brain. These studies are crucial for developing targeted dietary interventions for specific populations.

### 2.4 Ketogenic diet

The ketogenic diet (KD) is defined by its low carbohydrate and high fat content, facilitating energy production through the enhancement of ketone body synthesis. Research conducted on animal models has demonstrated that this dietary approach can diminish microglial activation and neuroinflammation, leading to improvements in pathological alterations and cognitive performance in mice with AD (81). For older adults experiencing cognitive decline, following a ketogenic diet can be quite challenging. BRANDT carried out a 12-week investigation into the Modified Atkins Diet (MAD) among individuals with mild to moderate AD. The findings indicated that the generation of ketone bodies within the body may improve episodic memory and self-reported cognitive vitality in patients with early-stage AD (82). Nevertheless, a higher consumption of dietary fats could elevate concentrations of low-density lipoprotein (LDL) cholesterol and triglycerides in the bloodstream, potentially harming the cardiovascular and cerebrovascular systems while hastening cognitive deterioration (83). This appears to be at odds with the cognitive advantages associated with ketone bodies in the ketogenic diet, which serve as an alternative energy substrate for the brain. OLSON suggested that the ketogenic diet could worsen cognitive deficits caused by intermittent hypoxia in murine models (84). Human studies indicated that this dietary pattern may negatively impact the gut microbiome, leading to a decrease in its overall diversity. In children with epilepsy, the ketogenic diet has been shown to reduce the relative abundance of *Bifidobacterium*, *Eubacterium rectale*, and Dialister, while increasing the relative abundance of *Actinobacteria* and *Escherichia coli* (19).

Consequently, future investigations should prioritize the safety and tolerability of the ketogenic diet among elderly patients to ensure that its implementation does not impose an excessive burden. Considering the potential adverse effects associated with the ketogenic diet, some researchers have suggested a modified Mediterranean ketogenic diet, which integrates essential components of both the Mediterranean and ketogenic dietary frameworks. A randomized trial conducted in 2021 demonstrated that this hybrid diet can enhance patients’ daily functioning and overall quality of life; however, additional trials are necessary to further substantiate its efficacy (85).

### 2.5 Other dietary patterns

An increasing body of research indicates that both the Dietary Approaches to Stop Hypertension (DASH) and ketogenic diets positively influence cognitive health. The DASH diet is acknowledged as an effective non-pharmacological intervention for hypertension. Its nutritional framework prioritizes a well-rounded diet abundant in fruits, vegetables, whole grains, low-fat dairy, and lean proteins. Research has demonstrated that among older adults, sustained adherence to the DASH diet correlates with enhanced cognitive function (86). The unsaturated fatty acids, antioxidants, and polyphenols present in the DASH diet have the potential to mitigate neuroinflammation in the brain that is associated with cognitive decline (87).

Current research findings, however, exhibit a lack of consistency. For instance, a randomized trial investigating cognitive impairment in adults indicated that the DASH diet alone did not yield cognitive advantages (88). Conversely, another study found no correlation between adherence to the DASH diet and improved cognitive health within a European demographic (89). Notably, a longitudinal study that analyzed 12 years of follow-up data determined that adherence to the Mediterranean-DASH Intervention for Neurodegenerative Delay (MIND) diet could potentially lower the risk of cognitive decline by 53% (90). In summary, the variability in study outcomes may be attributed to factors such as trial design, the assessment metrics employed to evaluate adherence to the DASH diet, and the variations in food selection across the studies.

Vegetarian diet, primarily composed of vegetables and fruits, excludes all types of meat, fish, and seafood (91). This dietary pattern has been shown to reduce the loss of dopaminergic neurons and improve negative emotional states and constipation symptoms in Parkinson’s disease (PD) patients (92). Research indicates a positive correlation between the abundance of Enterobacteriaceae and PD severity. PD patients exhibit increased levels of Akkermansia, *Lactobacillus*, and *Bifidobacterium*, along with reduced *Prevotellaceae* abundance (93, 94). Interestingly, vegetarians experience lower levels of stress and anxiety, exhibit better emotional well-being, and can effectively alleviate anxiety and depression in PD patients compared to meat-eaters, and vegetarians had higher abundance of certain *Bacteroidetes* in their guts, especially *Prevotella* (95). Fecal short-chain fatty acid levels were positively correlated with fruit, vegetable, and legume intake (96). A German case–control study comparing gut microbiota composition in PD patients and healthy individuals found that lacto-ovo vegetarian dietary interventions altered gut microbiota composition in PD patients (97). Increasing evidence supports the hypothesis that inflammatory processes play a crucial role in PD and may represent a key mechanism within the gut-brain axis. Changes in the gut microbial metabolome may have direct or indirect effects on brain health and disease progression. As such, dietary interventions that shift gut microbiota composition hold promise as potential therapeutic strategies to influence disease progression and alleviate symptoms in neurodegenerative disorders like PD.

**Figure 1:**
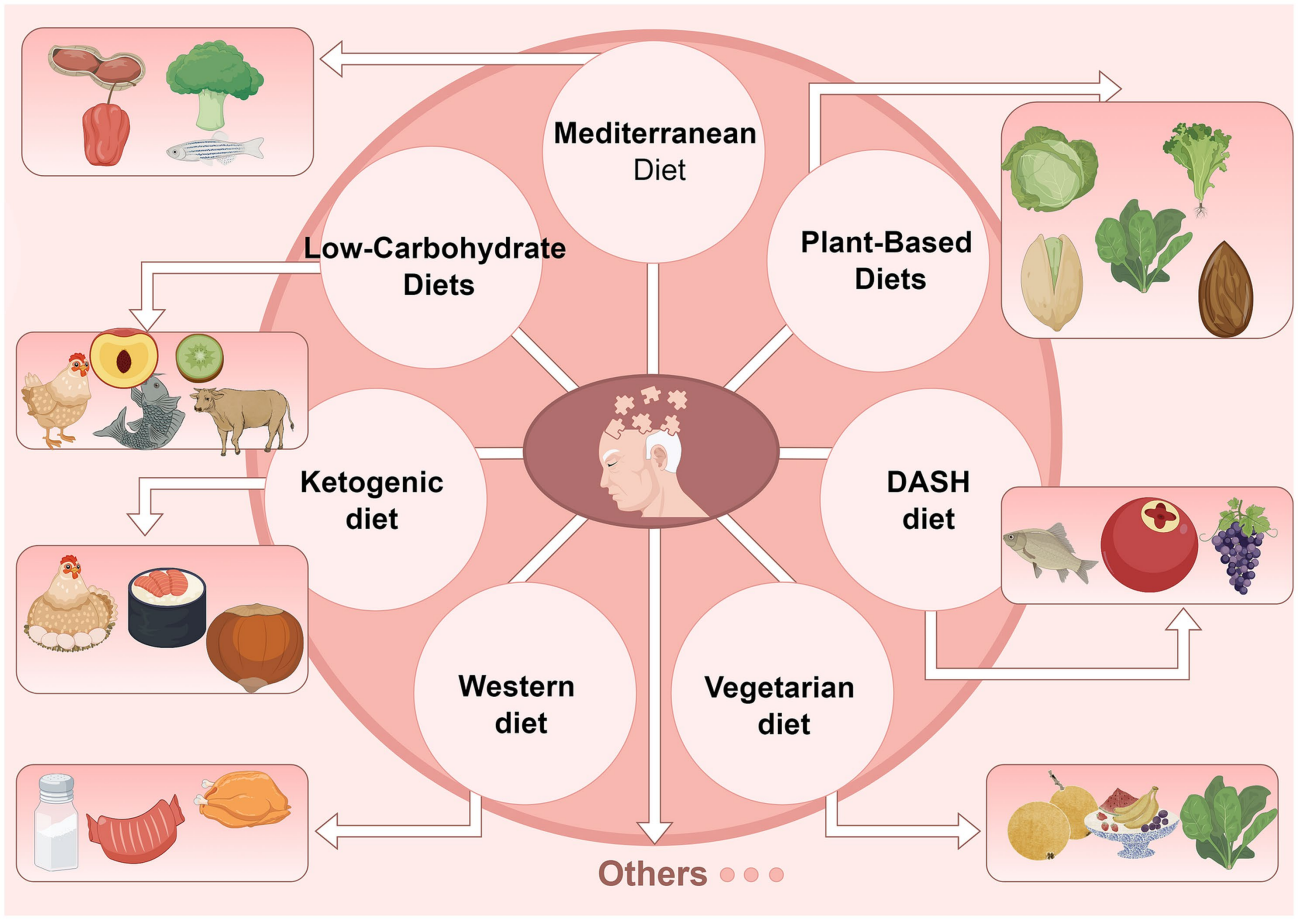
Different dietary patterns affect cognitive function.

The Western diet, commonly observed in developed Western countries, is characterized by a low intake of grains and a high consumption of animal-based foods and added sugars, often lacking in fiber, vitamins, and minerals (98). High-calorie intake associated with this dietary pattern can harm central dopaminergic neurons through neurotoxic effects. Additionally, the heme iron abundant in red meat, a staple of the Western diet, contributes to oxidative stress and increases the risk of PD (99). A systematic review has revealed that ultra-processed foods (a hallmark of the Western diet) disrupt gut microbiota, damage the nervous system, and promote the development and progression of neurodegenerative diseases, including PD (100). Although traditional Chinese dietary habits differ from the Western diet, western influences have led to increased consumption of ultra-processed and high-sugar foods in recent years. This shift has correlated with rising rates of PD, anxiety, and depression in the population, highlighting the need for greater attention to dietary habits and their long-term impacts on health. In conclusion, we present a summary of the dietary pattern in Figure 1.

## 3 Effects of nutrients on cognitive function

Numerous studies suggested that various dietary patterns can be effective in preventing or treating cognitive health issues. However, considering the synergistic or antagonistic interactions among different nutrients, one limitation of altering entire dietary approaches is identifying which specific nutrients are associated with cognitive improvement. Consequently, increasing attention is being directed toward the effects of individual nutrients on cognitive function. In this regard, we will discuss their impacts on cognitive improvement from the perspectives of polyunsaturated fatty acids, vitamins, polyphenols, and dietary fiber.

### 3.1 Polyunsaturated fatty acids

Long-chain polyunsaturated fatty acids (LC-PUFAs) encompass both ω-6 and ω-3 fatty acids, with the latter being the focus of more comprehensive research concerning cognitive function. The principal ω-3 fatty acids are eicosapentaenoic acid (EPA) and docosahexaenoic acid (DHA). G-protein coupled receptors (GPCRs), situated on neuronal membranes within the brain, function as receptors for neurotransmitters such as dopamine and serotonin (101). The role of ω-3 fatty acids is intricately linked to GPCR functionality, underscoring their importance for the proper operation of neural tissues. The primary source of ω-3 fatty acids for the human body is through dietary intake. Prolonged dietary insufficiencies of ω-3 fatty acids can disrupt normal cognitive processes, resulting in cognitive deterioration, memory impairments, and compromised spatial navigation abilities. Furthermore, such deficiencies may correlate with various neurological conditions, including mood disorders and dementia. The World Health Organization (WHO) advises a daily consumption of 500 mg of EPA and DHA, which corresponds to the quantity present in approximately 50 grams of salmon (102).

The findings indicated that a decreased consumption of ω-3 and ω-6 fatty acids could be linked to cognitive decline; however, no significant correlation was observed between variations in the ratio of these two fatty acids and cognitive function (103). A comprehensive review examined the association between ω-3 fatty acids and cognitive function in older adults with differing initial cognitive conditions (104). The findings revealed that in 10 of the 14 randomized controlled trials (RCTs) analyzed, supplementation with ω-3 fatty acids enhanced specific cognitive domains in the elderly, including working memory, executive function, verbal memory, short-term memory, and perceptual speed. This indicates that ω-3 fatty acid supplementation exerts a beneficial influence on cognitive functions in older adults. Furthermore, the cognitive effects of ω-3 supplementation may be associated with the initial cognitive status of the elderly population. Clinical trials evaluating the impact of LC-PUFA on cognitive performance may be affected by variables including the specific type and origin of fatty acids, as well as the initial cognitive condition of the participants (105). Consequently, further investigation is essential to gain a deeper insight into the impact of LC-PUFA on cognitive deficits associated with Alzheimer’s disease.

The connection between the consumption of dietary *ω*-3 fatty acids and cognitive health remains incompletely elucidated; however, various mechanisms have been suggested: (1) Antioxidant and Anti-inflammatory Effects: ω-3 polyunsaturated fatty acids, especially DHA and EPA, demonstrate significant antioxidant and anti-inflammatory effects. These compounds can impede lipid peroxidation in the brain and neuronal apoptosis, thereby playing a role in cognitive protection (106–108). (2) Immune Response Modulation: DHA and EPA contribute to immune responses by suppressing genes associated with inflammation. This modulation may aid in diminishing inflammatory processes within the brain that could hinder cognitive function. (3) Cell Membrane Composition: By substituting ω-6 polyunsaturated fatty acids and cholesterol, ω-3 PUFAs modify the composition of cell membranes, resulting in alterations in lipid raft clustering and influencing cellular signaling mechanisms. (4) Vascular Regulation: Certain derivatives of DHA and EPA function as vasoactive agents that assist in the regulation of cerebral perfusion, thereby potentially improving blood circulation to the brain. (5) Specialized Pro-resolving Mediators (SPMs): ω-3 polyunsaturated fatty acids are metabolized into SPMs, which exhibit anti-inflammatory and pro-resolving characteristics. SPMs facilitate the restoration of homeostasis by effectively orchestrating inflammatory responses, downregulating pro-inflammatory cytokines, and enhancing the expression of anti-inflammatory cytokines. They encourage the phagocytosis of cellular debris and apoptotic cells without inducing immune suppression, while also competing with pro-inflammatory eicosanoids derived from ω-6 fatty acids (106, 108–110). (6) Neurotransmitter Modulation: Recent studies suggest that the acute administration of EPA improves γ-aminobutyric acid (GABA) transmission through the modulation of serotonin 6 receptors, which may play a role in addressing learning and memory deficits observed in both adult and adolescent mice (111, 112).

Although ω-3 polyunsaturated fatty acids may mitigate cognitive decline via their anti-inflammatory properties, certain research indicates that dietary interventions may not lead to substantial enhancements in cognitive deficits (113). Furthermore, a study conducted by Pinelopi et al. suggests that administering high doses of ω-3 and ω-6 fatty acid supplements, alongside antioxidant vitamins, may enhance cognitive performance in older adults experiencing mild cognitive impairment (114). Nevertheless, the pathways through which ω-6 fatty acids exert their effects are not yet fully understood, underscoring the necessity for additional investigations to elucidate the connection between ω-6 fatty acid consumption and cognitive function.

### 3.2 Vitamins

#### 3.2.1 Vitamin B

B vitamins play a crucial role in numerous vital physiological functions, including the synthesis and repair of DNA, RNA, proteins, and phospholipids, as well as the methylation cycle, nutrient metabolism, cellular metabolism and repair, and energy generation. Some studies indicate that B vitamin supplementation may offer significant neuroprotective benefits. Vitamin B1 is a critical nutrient for brain metabolism, cellular function, and the production of neurotransmitters, such as acetylcholine. A deficiency in this vitamin can result in disruptions in oxidative metabolism, neuroinflammation, endoplasmic reticulum stress, autophagy, and neurodegenerative processes (115). Numerous studies indicate that elevated levels of vitamin B1 and its analogs may mitigate Alzheimer’s disease-associated pathological alterations; however, further clinical evidence is required to substantiate this perspective (116). Furthermore, vitamins B2 and B5 play a role in oxidative processes; however, the debate continues regarding their potential to mitigate age-related cognitive decline through the reduction of oxidative damage. Vitamin B3 serves as a precursor to the coenzyme nicotinamide adenine dinucleotide (NAD). Preliminary research indicates that NAD and its precursors may contribute to the preservation of normal cognitive function across different pathological conditions (117). Research findings show that vitamin B3 interacts with the specific receptor hydroxycarboxylic acid receptor 2 (HCAR2), leading to a decrease in amyloid plaque load and neuritic dystrophy in a mouse model of Alzheimer’s disease (5x FAD mice) (118). Choline, known as vitamin B4, plays a crucial role in the management of patients with Alzheimer’s disease, with cholinesterase inhibitors being a primary pharmacological intervention employed in the treatment of AD (119).

Folic acid (vitamin B9), vitamin B6, and vitamin B12 are essential cofactors crucial in the one-carbon metabolism pathway, responsible for producing and transporting organic groups with a single carbon atom. The impact of these B vitamins on cognitive impairment associated with Alzheimer’s disease is linked to their function in the methionine cycle. Insufficient levels of these vitamins may result in the buildup of homocysteine (Hcy), leading to reduced S-adenosyl methionine (SAM) levels. A clinical trial demonstrated that daily intake of 0.8 mg folic acid, 0.5 mg vitamin B12, and 20 mg vitamin B6 could effectively reduce Hcy levels and decelerate the progression of mild cognitive impairment (MCI) in patients (120). Elevated levels of Hcy have been linked to an increased risk of AD in older individuals (121). Nevertheless, a study conducted by Kwok et al. revealed that providing daily doses of 0.5 mg vitamin B12 and 0.4 mg folic acid to patients with MCI and high serum Hcy levels (≥10.0 mmol/L) did not result in enhanced cognitive function. Instead, the supplementation only temporarily alleviated depressive symptoms in MCI patients (122). Additionally, the researchers identified a potential interaction between aspirin and B vitamins.

#### 3.2.2 Vitamin D

Vitamin D is a crucial nutrient for the human body and is a fat-soluble vitamin primarily acquired through skin synthesis and dietary consumption. Within the body, vitamin D is present in two primary forms, vitamin D2 and vitamin D3, with vitamin D3 constituting the majority (90–95%) of the total vitamin D content. Both forms of vitamin D are biologically inert and require hydroxylation in the liver and kidneys to be transformed into their active forms, 1,25-dihydroxyvitamin D2 and 1,25-dihydroxyvitamin D3, which then exert their physiological effects (123). Vitamin D is crucial not only for calcium and phosphorus metabolism, immune regulation, and anti-inflammatory processes, but also for various brain functions such as neuroimmune regulation, neurotrophic factor modulation, neuroprotection, neuroplasticity, and brain development (124–127). Recent studies have increasingly connected vitamin D to neuropsychiatric conditions, with research indicating a link between vitamin D deficiency and disorders like tic disorders and cognitive impairment (128–132).

Numerous studies have identified a link between inadequate vitamin D levels and depression. In a prospective investigation by Ronaldson and colleagues, involving 127, 244 middle-aged participants from the UK Biobank, it was observed that individuals with insufficient or deficient vitamin D levels exhibited a higher susceptibility to developing depression (133). Briggs conducted a study involving 3,965 individuals aged 50 and above residing in the community, revealing that those with insufficient levels of vitamin D (<30 nmol/L) exhibited a notably increased susceptibility to depression onset (134). The research involving 186 individuals with gout revealed that 32 of them (17.2%) were found to have depression. Interestingly, it was observed that these patients exhibited notably lower levels of 25-hydroxyvitamin D in comparison to those who did not experience depression (135). Research has indicated a correlation between vitamin D levels and cognitive function among individuals suffering from depression. Those with depression and lacking sufficient vitamin D are at a higher risk of experiencing cognitive deficits, which can impact their overall well-being. Moreover, studies have shown a potential association between vitamin D levels and the development of AD, with severe vitamin D deficiency (<10 ng/mL) significantly increasing the likelihood of dementia and AD onset (136, 137). Attention deficit and hyperactivity disorder (ADHD), a prevalent chronic neurodevelopmental disorder affecting approximately 7.2% of children globally, has been the focus of research by Thomas et al. (138) and Li et al. (139). A case–control study conducted by the researchers revealed that 52.4% of children diagnosed with ADHD had a vitamin D deficiency (defined as <20 μg/L). The study also highlighted a negative correlation between vitamin D levels and the overall ADHD screening scale score, particularly in the inattention subscale. Furthermore, a randomized double-blind controlled trial illustrated that vitamin D supplementation led to a significant improvement in impulsive behavior among children with ADHD (140). These results underscore the potential link between vitamin D levels and ADHD development, suggesting that vitamin D supplementation could serve as an innovative approach to ameliorate clinical symptoms in affected children.

#### 3.2.3 Antioxidant vitamins

Common antioxidant vitamins such as vitamin A, vitamin C, vitamin E, and beta-carotene are believed to play a crucial role in protecting the brain from oxidative damage. Some researchers suggest that oxidative stress or insufficient antioxidant defense might contribute to the development and progression of dementia. An 8-year vitamin supplementation trial demonstrated sustained cognitive benefits, including improved episodic and verbal memory even 6 years after the trial’s conclusion, particularly evident in individuals with lower baseline antioxidant levels (141). However, a separate randomized controlled trial involving 2,824 women with cardiovascular disease or at risk of cardiovascular disease showed that supplementation with vitamins A, C, and E did not halt cognitive decline in these women, highlighting uncertainty regarding the impact of vitamin supplementation on cognitive function (142). Furthermore, various cross-sectional studies and intervention trials have provided evidence supporting the beneficial effects of dietary intake or supplementation of antioxidant vitamins on cognitive function (143, 144).

### 3.3 Polyphenols

Polyphenols constitute a vast category of phytochemicals prevalent throughout the plant kingdom, encompassing around 8,000 distinct structures. These compounds are identified by the presence of one or more aromatic rings with hydroxyl groups and are frequently present in fruits, vegetables, whole grains, olive oil, and green tea (145). Recent studies indicate that flavonoids could regulate signaling pathways associated with cognitive and neuroprotective functions, including the inhibition of acetylcholinesterase, butyrylcholinesterase, tau protein aggregation, and β-secretase. This modulation contributes to the deceleration of Alzheimer’s disease progression. Furthermore, dietary polyphenols are essential for their anti-inflammatory properties, ability to diminish oxidative stress, safeguard endogenous compounds from oxidative harm, regulate metabolism, and enhance endothelial and platelet function (146). Several research studies have indicated that certain refined polyphenols such as curcumin, EGCG from green tea, cyanidin, resveratrol, and tannic acid have the potential to alleviate age-related cognitive decline (147–149). SHISHTAR conducted a study that examined the correlation between long-term dietary flavonoid intake and cognitive impairment in humans, suggesting that a diet high in flavonoids could lower the risk of developing Alzheimer’s disease (150). Additionally, a prospective placebo-controlled trial demonstrated that supplementation with freeze-dried grape powder rich in polyphenols could help reduce brain pathology in individuals with mild cognitive impairment and offer protection against cognitive decline23. Kaplan and colleagues also found that a diet rich in polyphenols offers neuroprotective benefits against age-related brain atrophy (151). The cognitive advantages associated with polyphenols are believed to stem from the combined effects of diverse phenolic compounds thanks to their potent antioxidant properties. Studies have confirmed that natural antioxidants, such as polyphenols, can modulate gut microbiota, opening new avenues for their application in patients with mild cognitive impairment (MCI), the prodromal stage of Alzheimer’s disease (152).

### 3.4 Dietary fiber

Dietary fiber (DF) refers to a class of carbohydrate polymers with a degree of polymerization ≥3 that cannot be digested or absorbed in the small intestine. Naturally occurring DF is diverse, with cereals providing DF primarily from bran or rice husk, including arabinoxylan, β-glucan, resistant starch, hemicellulose/lignin, among others. DF directly supplies energy and nutrients to gut microbiota, increasing the diversity and abundance of beneficial gut microbes, enhancing gut immune metabolism, and ameliorating cognitive impairment (153). Research indicates that specific dietary fibers and other components can improve gut health and function by increasing concentrations of beneficial metabolites like short-chain fatty acids (SCFAs). These metabolites exert anti-inflammatory effects, mitigating neuroinflammation (154, 155). Furthermore, DF improves cognitive impairment by promoting anti-inflammatory and beneficial gut bacteria, such as Bifidobacterium and Lactobacillus, which modulate neurotransmitter levels and the immune system, directly influencing brain function and emotional states (153). Shi et al. found that a chronic dietary fiber deficiency (FD) diet induces gut dysbiosis (Bacteroides and increased Proteobacteria), leading to neuroinflammation and synaptic engulfment by microglia in the hippocampus, which subsequently causes systemic neuroinflammation and cognitive deficits (156). Therefore, DF intake is closely associated with gut barrier integrity, metabolic regulation, and immune function, playing a significant role in mitigating neuro-metabolic disorders, and preventing neurodegenerative diseases such as depression and Alzheimer’s disease. In addition, there are other nutrients that also have a significant impact on cognitive function, which we summarize in Table 1.

**Table 1:** The effects of nutrients on cognitive function.

Nutrients	Influence	Mechanisms and effects
Vitamin B	Improves memory and cognition	Participate in neurotransmitter synthesis, promote energy metabolism, and support neuronal function
Vitamin C	Improve cognitive function	It has antioxidant effects, protects neurons, and reduces oxidative stress
Vitamin E	Reduces the risk of cognitive impairment	Antioxidant, reduce Aβ protein deposition, improve neurological health
Omega-3 fatty acids	Improves memory and learning	Promote neuron growth, strengthen neural connections, and improve brain function
Iron	Maintain brain energy metabolism	Involved in oxygen transport and neurotransmitter synthesis, a deficiency can affect cognitive abilities
Magnesium	Improves learning and memory	Participate in nerve signal transmission, regulate nerve excitability
Zinc	Promote neuroprotection	Involved in neurotransmitter synthesis, support neuron growth and repair
Complex carbohydrates	Improve cognitive ability	Stabilizes blood sugar levels, provides sustained energy, and supports brain function
Saturated fatty acid	May reduce cognitive function	Excessive consumption is associated with cognitive decline

## 4 Dietary intervention in the gut-brain axis improves cognitive function

The gut-brain axis serves as a two-way communication network that links the central nervous system (CNS) with the enteric nervous system (ENS) (157). The gut microbiota is essential for the synthesis of vitamins, amino acids, and peptides from undigested food in the gastrointestinal tract. They generate a range of secretions and microbial byproducts that boost immune responses, stimulate the release of cytokines, and regulate inflammatory processes. Moreover, the gut microbiota prevents protein decay and the proliferation of harmful microbes by metabolizing compounds like nitrosamines, hydrogen sulfide, and lactic acid (158, 159). An imbalance in the diversity and composition of gut microbiota is a significant contributor to the deterioration of gut integrity and functionality, resulting in heightened gut permeability, inflammation, and an altered gut environment. These alterations interfere with the communication pathways linking the gut and brain through neural connections, the hypothalamus-pituitary–adrenal axis, and the immune system, thereby triggering neurological dysfunction. Notably, cognitive function is particularly affected by these changes (160, 161). Dysregulation of the gastrointestinal system plays a role in the development and advancement of various diseases, such as neurological conditions (162). Research has identified a robust correlation between the gut-brain axis and cognitive performance. Empirical evidence shows that experimental mice deprived of gut microbiota display marked cognitive impairments (163).

**Figure 2:**
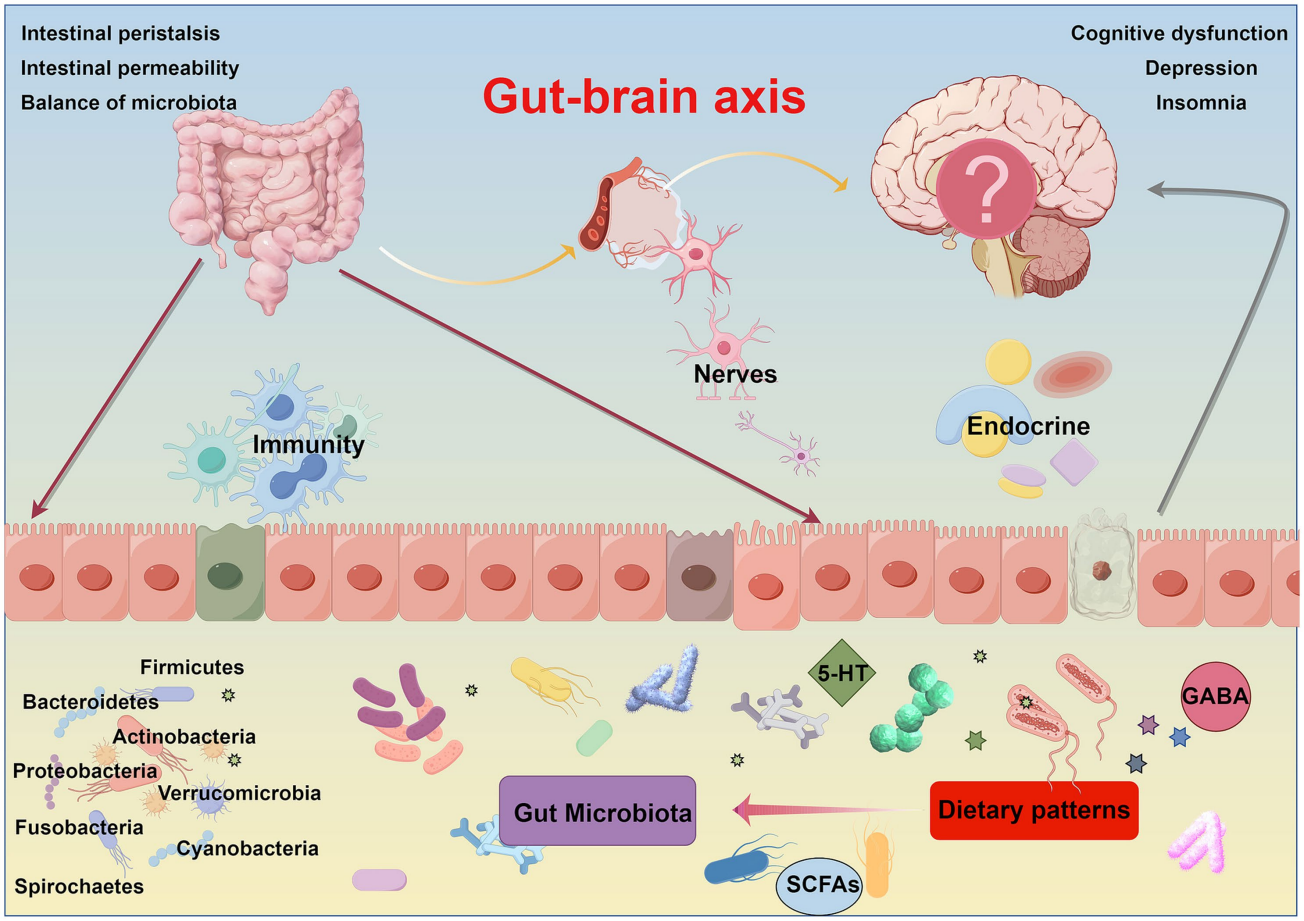
Dietary patterns affect cognitive function through the gut-brain axis.

Dietary interventions play a crucial role in enhancing neurodegenerative conditions and are strongly correlated with relevant risk factors. Research studies indicate that consuming excessive saturated fatty acids can worsen neurodegeneration in AD and PD by heightening oxidative stress and lipid peroxidation. Furthermore, high-calorie diets have been linked to the early onset of Huntington’s disease (HD) (164–168). Increased consumption of saturated fats can lead to inflammatory responses, allowing immune cells to enter the central nervous system (169). Diets high in fat may disrupt the balance of gut microbiota, potentially causing neuropsychiatric issues (170). These diets can cause oxidative stress in the gut, leading to neuronal cell death and cognitive decline through the inactivation of the Nrf2 pathway. Furthermore, high-fat diets can increase the deposition of amyloid-beta, raising the risk of Alzheimer’s disease (171). Nutrients and their byproducts in the diet play a role in regulating neuroinflammation and improving brain function. Dietary changes can help manage metabolic and functional problems associated with neurodegenerative diseases. In contrast, the intake of DHA has been linked to a reduced risk of Alzheimer’s and Parkinson’s diseases. DHA not only influences gut microbiota and gene expression but also enhances communication between the gut and brain (172). Animal research has shown that enriching diets with ω-3 polyunsaturated fatty acids containing DHA can influence the composition of gut microbiota, leading to enhanced cognitive function and reduced anxiety-related behaviors in mice (173). Notably, the effects of DHA supplementation differ according to gender, with male mice experiencing more significant benefits compared to females. Furthermore, the KD, characterized by high fat and low carbohydrate intake, has been found to impact gut microbiota. In newborns, a week of KD has been shown to decrease harmful bacteria and increase beneficial microbes compared to infants following a typical diet (174). In children with epilepsy, long-term adherence to KD results in decreased gut microbiota diversity, lower levels of *Firmicutes*, and higher levels of *Bacteroidetes* (175). The ketogenic diet demonstrates neuroprotective properties through its influence on gut microbiota. Studies on animals reveal that KD promotes the growth of beneficial gut bacteria, enhances blood flow to the brain, and supports neurogenesis by activating endothelial nitric oxide synthase (eNOS) and inhibiting mTOR signaling (176). Moreover, the production of (D)-3-hydroxybutyrate ketone bodies induced by KD may facilitate communication between the gut and the brain via G protein-coupled receptor (GPCR) signaling pathways and the epigenetic regulation of relevant genes (Figure 2).

The diversity and integrity of gut microbiota play a crucial role in maintaining health, closely tied to normal bodily functions and changes related to disease, we summarize the main classifications and functions of the human gut microbiota in Table 2. Gut microbiota have a significant impact on advanced brain activities like cognition and emotion, and their imbalance can be a key factor in the development of neurodegenerative conditions. The complex gut environment is influenced by various internal and external factors, making gut microbiota both a source of stress and a potential target for intervention. Imbalance in gut bacteria can impair cognitive abilities and contribute to neurodegenerative diseases. Dietary habits influence the gut microbiota composition, subsequently impacting the interaction between the gut and the brain. This interaction boosts cognitive functions and mitigates symptoms associated with neurodegenerative conditions, as shown as Table 3. However, extended consumption of high-fat or high-sugar diets may harm cognitive function and worsen neurological conditions. Although the exact relationship between gut microbiota, cognitive function, and neurodegenerative diseases is not fully established, forthcoming research is expected to provide more insights into how personalized nutrition can leverage the gut-brain connection to support cognitive well-being and prevent neurodegenerative ailments, we summarized the relationship between intestinal flora and cognitive dysfunction in Table 4.

**Table 2:** Main classification and function of human gut microbiota.

Types of microorganisms	Key members	Main functions
Bacteria	Firmicutes, Bacteroidetes, Actinobacteria, Proteobacteria, Verrucomicrobia	Produce short-chain fatty acids, regulate the immune system, inhibit pathogen growth; synthesize vitamins, maintain intestinal barrier function, etc.
Fungi	*Candida albicans*, Aspergillus	Participate in the fermentation of carbohydrates; competing with bacteria for niche; Modulating immune response
Virus	Bacteriophages, Eukaryotic viruses	Regulates bacterial population dynamics, influences the evolution of bacterial genomes, and interacts with the host immune system
Protist	Amoebae, Flagellates	Involved in the ecological balance of the gut, interacting with bacteria and the host immune system
Archaea	Methanobrevibacter	It is involved in methane production and affects the intestinal fermentation process

**Table 3:** Effects of different dietary patterns on gut microbiota and cognition health.

Dietary patterns	Main features	Effects on the gut microbiome	Effects on the cognition health
Mediterranean diet	Rich in fruits, vegetables, whole grains, nuts, olive oil, and fish	Enhances diversity of gut microbiota; increases beneficial bacteria like Bifidobacteria and Lactobacilli; reduces inflammation	Improves cognitive function, reduces AD risk. Studies show benefits in Chile and among U.S., French, Spanish, and Greek elderly populations. EVOO and moderate wine intake linked to cognitive benefits. Dairy improves omega-3:omega-6 ratio, reducing inflammation
Plant-based diets	Rich in Plant-based foods and minimize or restrict animal-derived products	Increases fiber-degrading bacteria; promotes diversity and beneficial metabolites; reduces inflammation	Reduces dementia risk in Taiwanese vegetarians; inconsistent effects in U.S. cohort studies. Helps mitigate depressive symptoms in obese elderly and improves gut microbiota diversity
Low-carbohydrate diets	High in fat, moderate in protein and very low in carbohydrates	Reduces carbohydrate-fermenting bacteria; alters microbiota diversity; may favor growth of ketone-utilizing microbes	Improves cognitive function in obesity and diabetes-related impairments. Increases BDNF in hippocampus and prefrontal cortex
Ketogenic diet	Rich in low carbohydrate and high fat content	Decreases diversity; increases Akkermansia, which has anti-inflammatory properties; may disrupt long-term microbiota balance	Improves cognition in AD mice; Modified Atkins Diet showed memory benefits in early-stage AD patients. Suggested hybrid Mediterranean-Ketogenic Diet offers cognitive and functional benefits
Dietary approaches to stop hypertension (DASH) diets	High in fruits, vegetables, whole grains, low-fat dairy	Supports gut microbiota diversity; increases bacteria associated with fiber fermentation and short-chain fatty acid production	Enhances cognitive health in elderly. MIND diet, a hybrid of DASH and Mediterranean diets, reduces cognitive decline risk by up to 53%. However, inconsistent effects across studies
Vegetarian diet	Free of meat and animal products and rich in fruits, vegetables, legumes, nuts and whole grains	Promotes beneficial bacteria like Lactobacillus and Bifidobacterium; decreases Prevotellaceae, linked to inflammation	Alleviates anxiety, depression, and PD symptoms. Alters gut microbiota composition in PD patients, supporting gut-brain axis modulation
Western diet	High in fat, sugar and salt	Reduces beneficial bacteria; increases pro-inflammatory bacteria; disrupts gut barrier integrity	Associated with PD progression, gut microbiota disruption, and neuroinflammation. Linked to increased PD, anxiety, and depression in populations adopting Western dietary habits

**Table 4:** The relationship between gut microbiota and cognitive dysfunction.

Name of disease	Changes in gut microbiota
Alzheimer’s disease	Gut microbial diversity decreased significantly, with an increase in Bacteroidetes, a decrease in Firmicutes, a decrease in beneficial bacteria (such as Lactobacillus and bifidobacterium), and an increase in bacteria associated with inflammation (such as Clostridium)
Parkinson’s disease	Gut microbiota composition differed from healthy controls, with a decrease in beneficial bacteria such as Lactobacillus and Bifidobacterium, an increase in bacteria associated with inflammation and neurodegeneration (such as Proteobacteria), and a significant decrease in Prevotellaceae
Autism spectrum disorder	The diversity of gut microbes has decreased, with a decrease in the genera Prevotella and Faecalibacterium, an increase in the genera Clostridium and Desulfovibrio, and an abnormal proportion of bacteria related to neurotransmitter metabolism
Mild cognitive impairment	Intestinal microbial diversity decreased, Bacteroidetes increased, Firmicutes decreased, short-chain fatty acid (SCFAs) producing bacteria decreased, and bacteria associated with inflammation increased
Multiple sclerosis	Intestinal microbial diversity decreased, with an increase in Bacteroidetes, a decrease in Firmicutes, a decrease in bacteria associated with immune regulation (such as Ackermannia), and an increase in bacteria associated with inflammation (such as Proteobacteria)
Depression	Gut microbial diversity decreased, bacteria associated with inflammation (such as clostridium) increased, beneficial bacteria such as Lactobacillus and Bifidobacterium decreased, and short-chain fatty acid (SCFAs) producing bacteria decreased
Anxiety disorder	The composition of the gut microbiome is abnormal, with a decrease in bacteria associated with neurotransmitter metabolism (such as lactobacillus) and an increase in bacteria associated with inflammation

## 5 Dietary recommendations for gut-brain axis treatment of cognitive dysfunction

Numerous epidemiological studies indicate a correlation between diet and cognitive function, with healthy dietary habits serving as a protective factor for cognitive well-being. Hence, improving the quality of life for elderly individuals with cognitive impairments necessitates the development of a tailored dietary health management plan that aligns with the lifestyle practices of Chinese residents. This discussion will delve into two key components: dietary patterns and essential nutrients.

Among various dietary schemes, the Mediterranean diet, plant-based diet, and low-carbohydrate diet exhibit protective properties against cognitive decline and mild cognitive impairments in the elderly. However, it is crucial to acknowledge the dual nature of the ketogenic diet in relation to the potential development of eating disorders. Moreover, findings from different studies present inconsistencies, possibly influenced by research methodologies, assessment tools, and participant demographics. Therefore, future investigations should prioritize long-term, large-scale, multi-center randomized controlled trials to comprehensively assess the impact of diverse nutrients and dietary patterns on cognitive function.

Numerous animal experiments randomized controlled trials involving human subjects as participants, and the analysis of essential nutrients in humans have collectively revealed that *ω*-3 polyunsaturated fatty acids, B vitamins, polyphenolic compounds, and vitamin D are notably associated with cognitive protection. Specifically, ω-3 polyunsaturated fatty acids are known for their anti-inflammatory, immunomodulatory, and neuroprotective properties. The extensive body of evidence supports the notion that consuming adequate levels of ω-3 polyunsaturated fatty acids can enhance cognitive function significantly (177, 178). Recent research has indicated a strong correlation between the consumption of B vitamins and cognitive performance in individuals diagnosed with Alzheimer’s disease and those experiencing mild cognitive impairment (179). B vitamins exhibit promising therapeutic benefits in addressing vascular cognitive dysfunction and cognitive issues in individuals with diabetes and cerebral infarction when compared to conventional treatments. Additionally, the long-term consumption of polyphenolic compounds can aid in preventing cognitive decline among older individuals. Notably, research indicates that elderly individuals in India who incorporate curcumin-rich diets have a significantly lower dementia rate, around 73%, in comparison to their counterparts in the United States. Furthermore, Vitamin D, a fat-soluble vitamin, plays a crucial role in enhancing calcium and phosphorus absorption in the small intestine while also supporting skin cell growth, immune function regulation, and differentiation. Food sources rich in Vitamin D include fatty marine fish, animal liver, egg yolk, butter, and cod liver oil. It is important to note that while single nutrient supplementation or multi-component supplements may yield conflicting outcomes, the interplay of various food components in a balanced diet is key to promoting health. The synergy between phytochemicals from fruits and vegetables combined with fatty acids from fish, as well as the mutual absorption of different nutrients when consumed together, underscores the importance of considering the overall dietary pattern rather than focusing solely on individual nutrients.

Current research, both domestic and international, has made significant progress in understanding the dietary needs of elderly individuals with cognitive impairment. There is a substantial body of evidence indicating that modifications in diet and nutrition play a crucial role in preserving cognitive function, emotional well-being, immune response, and vascular health. While early dietary adjustments tend to yield more favorable outcomes, adopting health-promoting behaviors at any stage of life can enhance longevity and overall well-being (180). We generally recommend adopting a well-rounded and moderate diet, adjusting the intake of carbohydrates and fats, and ensuring adequate consumption of vitamins and minerals as a potential optimal approach to preventing or enhancing cognitive function.

## 6 Discussion

Recent studies have extensively investigated the effects of dietary interventions on cognitive well-being. Notable diets such as the Mediterranean diet, plant-based diets, and low-carbohydrate diets have shown protective advantages for cognitive function. These dietary patterns are rich in antioxidants, anti-inflammatory agents, and neuroprotective nutrients like polyunsaturated fatty acids, B vitamins, polyphenols, and vitamin D. Research suggests that these nutrients may decelerate cognitive decline and reduce the risk of neurodegenerative conditions by addressing inflammation, lessening oxidative stress, and promoting neurogenesis, among other mechanisms.

However, despite the potential benefits of dietary interventions for cognitive health, several obstacles impede their successful implementation. Challenges include difficulties in maintaining adherence to specific dietary patterns over time and variations in individual responses to dietary changes across different populations, necessitating personalized dietary guidance. Moreover, factors such as socioeconomics, cultural influences, and resource availability also impact the effectiveness of dietary interventions.

**Figure 3:**
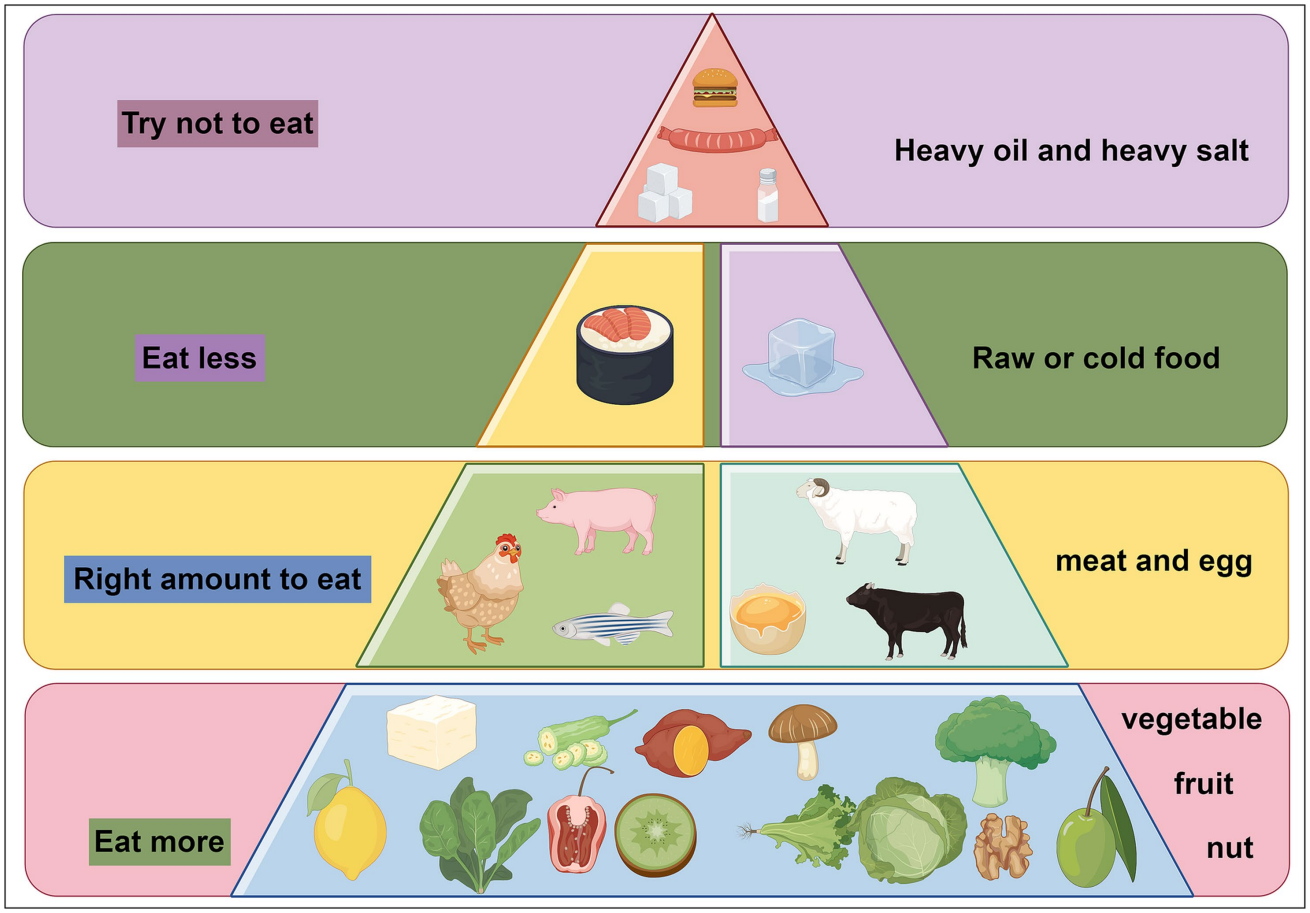
Dietary recommendations for cognitive dysfunction. We recommend eating more fresh fruits and vegetables, eating a moderate amount of meat, eating less raw and cold food, and trying not to eat foods high in salt, sugar, and fat.

Drawing from existing literature, we recommend various dietary strategies to improve cognitive health. These include emphasizing the consumption of fruits, vegetables, whole grains, nuts, and healthy fats such as olive oil, while moderating fish and meat intake. Increasing the intake of vegetables, fruits, whole grains, legumes, nuts, and seeds while reducing animal product consumption is also beneficial. Additionally, limiting high-carbohydrate foods like sugar, bread, and pasta while increasing fat and protein intake is advised. Supplementing with polyunsaturated fatty acids, B vitamins, polyphenols, vitamin D, and essential nutrients is also suggested, as shown as Figure 3.

To enhance our comprehension of how dietary interventions impact cognitive health, future research should adopt a multidisciplinary approach involving nutrition, neuroscience, psychology, and sociology to comprehensively evaluate the effects of dietary modifications. Long-term, large-scale, multi-center randomized controlled trials are essential to explore the sustained impacts of diverse dietary patterns on cognitive performance. Analyzing existing dietary frameworks can aid in developing personalized dietary recommendations tailored to individual variances, thereby enhancing adherence and efficacy of interventions. Furthermore, examining the influence of socioeconomic and cultural factors on dietary strategy implementation is crucial for establishing more effective public health initiatives. Through these holistic endeavors, we can optimize dietary interventions to prevent and alleviate cognitive impairment, ultimately enriching individuals’ quality of life.

## 7 Conclusion

With the advancement of human gut microbiota analysis technologies, the critical role of microorganisms has become increasingly evident. The brain and gut interact through the microbiota-gut-brain axis, regulating brain functions and the progression of cognitive impairments. The gut microbiota, as a dynamic ecosystem, undergoes continuous changes influenced by internal and external environmental factors. Dysbiosis of the gut microbiota may serve as both a stressor and an intervention target for neurodegenerative diseases, as it can induce cognitive dysfunction and contribute to these conditions. Dietary modifications and the broad intake of nutrients can mediate gut-brain communication by regulating the gut microbiota, thereby improving cognitive function, and alleviating neurodegenerative diseases. However, the causal relationship between dietary habits, gut microbiota, and the pathology of cognitive functions has yet to be fully established. More extensive and in-depth research is needed to clarify how different dietary patterns influence the gut microbiota and their impact on cognition and neurodegenerative diseases. Importantly, improving dietary structures and adjusting the quantity, types, and distribution of gut microbiota to address cognitive dysfunction may become a novel therapeutic strategy in the future.
